# Ensuring the Efficiency and Effectiveness of Joint Clinical Assessment in National HTA Decision-Making: Insights from the 2024 CIRS Multi-Stakeholder Workshop

**DOI:** 10.3390/jmahp13010009

**Published:** 2025-03-03

**Authors:** Ting Wang, Neil McAuslane

**Affiliations:** Centre for Innovation in Regulatory Science (CIRS), London EC3A 8BE, UK; nmcauslane@cirsci.org

**Keywords:** Health Technology Assessment, joint clinical assessment, performance metrics, patient access

## Abstract

Background: This study explored the readiness and strategic considerations of companies and key stakeholders for the implementation of the Joint Clinical Assessment (JCA) under the European Health Technology Assessment Regulation (HTAR). It examined the implications of the JCA process for jurisdictional submission strategies, and decision-making across Europe. The study aimed at identifying key measures for an efficient and effective JCA process to enable national rollout. Methods: A survey was conducted with international pharmaceutical companies, followed by a multi-stakeholder workshop that expanded on the findings. The survey and workshop focused on key areas such as time to market, submission strategies, and the role of JCA in national decision-making processes. Descriptive and qualitative analyses were performed to identify recommendations for measuring and improving the JCA process. Results: 13 companies responded to the survey, respondents were generally prepared for the JCA process (readiness rated 6–7/10), but concerns persist about timeline uncertainties and timely JCA report delivery. In the short term, success for the HTAR from the company perspective is measured by positive recommendations across EU jurisdictions. Long term, the focus shifts to aligning HTA methodologies and evidence requirements across the EU. Establishing metrics to assess the efficiency and effectiveness of the JCA is a key step in the HTAR’s ongoing learning journey. To enhance the efficiency of the JCA process, a list of metrics is recommended for continuous improvement, as well as establishing training programs to strengthen member states’ capabilities, fostering open dialog for sharing technology-specific insights, and creating open-source tools to support companies. Additionally, research should be conducted to understand agencies’ expectations of the JCA and how they will use its reports, grouping agencies by archetype to identify trends. A key recommendation is the development of a product-based scorecard to evaluate JCA submissions and reviews from various perspectives, ensuring the process meets stakeholders’ needs and can be effectively utilized in national decision-making. Conclusions: The JCA process offers a significant opportunity to streamline HTA decision-making across Europe. This study highlights several key measures and consideration for its successful rollout, including the need for clearer communication about the role of JCA in national decisions, measurement of rollout time components, and the development of quality evaluation frameworks. A collaborative, iterative approach, where stakeholders continually refine the system, will be essential for its effectiveness. Addressing these challenges will enable the JCA to enhance efficiency, consistency, and ultimately improve access to treatments for patients.

## 1. Background

The Regulation (EU) 2021/2282 on Health Technology Assessment (HTAR) marks a significant milestone in harmonizing clinical assessments for HTA decision-making across EU member states [1]. Its primary goals are to enhance patient access to innovative health technologies, improve the quality of HTA processes, and reduce redundancies for national HTA agencies and industry stakeholders. The HTAR includes joint clinical assessments (JCA), joint scientific consultations (JSC), the identification of emerging health technologies, and opportunities for voluntary cooperation.

To support the implementation of HTAR, set to commence in January 2025, an HTA Coordination Group and four subgroups—comprising representatives from member states’ HTA agencies—have been established. These groups are tasked with developing methodological and procedural guidance, building on the foundation laid by EUnetHTA21 [2]. By December 2024, four implementation acts had been released, detailing rules for the JCA of medicinal products, information exchange with the European Medicines Agency (EMA), conflict of interest management, and JSC procedures for medicinal products [3]. In addition to active cooperation at the European level, HTA agencies in member states are preparing for JCA implementation through activities such as horizon scanning, assessor training workshops, and internal restructuring. However, there are notable divergences in readiness among member states, particularly in jurisdictions with smaller or newer agencies. The national process between 2025 and 2030 will be complex, as agencies will need to operate dual processes (JCA and non-JCA) to align with HTAR’s rolling implementation timeline. Meanwhile, companies are proactively preparing for the upcoming JCA in 2025, both internally and externally, focusing on key issues such as evidence requirements and analytical methodologies [4,5].

The JCA follows a PICO (Population, Intervention, Comparator, Outcomes) framework. The process begins with a PICO survey sent to all 27 EU member states, each of which reports the PICOs relevant to its decision-making. These reported PICOs are then consolidated by two rapporteurs during the scoping phase. Once the PICO survey and consolidation are complete, the manufacturer prepares the JCA dossier [3]. While stakeholders are preparing for HTAR’s implementation in the coming weeks, most discussions are centered on the JCA. However, many questions remain regarding the practical application of JCA outputs in national reimbursement decision-making. Under HTAR, national HTA agencies are not obligated to use the JCA output but can reference it as a harmonized clinical evidence base while maintaining autonomy in national decision-making processes. Addressing these concerns will require a collective effort from both companies and agencies to ensure the JCA is practical and efficient in real-world contexts.

Numerous studies have been conducted to assess national-level HTA decision-making in Europe. For example, the CIRS annual reports compare HTA decisions in terms of timing and outcomes, highlighting the divergences in submission patterns to HTA agencies following EMA approval [6]. The EFPIA W.A.I.T. indicators evaluate the market access environment in Europe, focusing on the timing of the launch of new medicinal products and illustrating how the access landscape varies across jurisdictions [7]. Cowie et al. proposed several strategies to expedite HTA decision-making post-regulatory approval, including early scientific advice to optimize data generation, aligning regulatory and HTA processes, and increasing the use of real-world evidence [8]. In addition to timing measures, studies have compared HTA decisions to understand the rationale behind decision-making across different agencies, with a particular focus on rare-disease products, oncology drugs, and EMA conditional approvals [9,10,11,12,13,14,15]. These studies emphasize the need for alignment and optimization of HTA processes across Europe, in particular for innovative products, which was the primary goal of the HTAR. Therefore, to ensure the HTAR is fit for purpose, it is essential to go beyond discussions of JCA preparation and processes. As the first implementation act is now adopted, a definitive procedure and timeline for JCA submission and production at the European level become apparent. Therefore, ensuring the practical utilization of JCA reports in national reimbursement determinations emerges as a critical focal point, which requires a collective effort to ensure practicability and efficiency in real-world contexts from both companies and agencies.

The objective of this study is to identify key factors for the implementation and utilization of JCA and to provide recommendations for evaluating its efficiency and effectiveness. This is conducted through a company perception survey and a multi-stakeholder workshop involving the European regulator, HTA agencies, industry representatives, patients, and academia.

## 2. Methods

The study consists of a company-driven survey followed by a multi-stakeholder workshop.

### 2.1. Perception Survey

A qualitative opinion survey was conducted to assess companies’ actions in preparing for JCA and their strategies for effectively rolling out medicines to national HTA agencies. A pilot version of the survey was developed in March 2024 by the first author and reviewed by potential responders from two invited pharmaceutical companies in April 2024. The pilot aimed to validate the clarity, format, and applicability of the survey. Feedback from the two independent responders was used to refine the wording of the questions and finalize the survey. The final survey included 11 multiple-choice, closed-ended questions, structured across three sections:

- Section 1 assessed companies’ current readiness for the HTA Regulation (5 questions).
- Section 2 explored companies’ preparedness for JCA and jurisdictional submissions (3 questions).
- Section 3 gathered insights on future improvements and adaptations based on current experiences and challenges (3 questions).

Each question included a free-text option for additional clarification or comments. The survey was distributed by email on 29 April 2024, to invited participants, who were asked to complete it by 6 June 2024. The questionnaire was sent to senior management at twenty international pharmaceutical companies, selected using purposive sampling from the membership of CIRS to ensure the study’s timeliness and maximize the response rate.

### 2.2. Multi-Stakeholder Workshop Discussion

A multi-stakeholder workshop was held on 14 June 2024, in Seville, Spain, on the topic of “Facilitating Joint Clinical Assessment (JCA) Implementation, Utilisation, and Timely Patient Access”. The workshop brought together 21 senior HTA/HEOR representatives from 18 international pharmaceutical companies, representatives from the EMA and 11 European HTA agencies, two patient representatives, and two academic representatives.

The survey results were presented at the meeting, followed by talks from keynote speakers, case studies, and breakout discussions. Participants were divided into two groups for the breakout sessions, with group 1 focusing on the efficiency of JCA, which is defined as the time taken between a new medicine’s submission to EMA to obtaining the initial HTA recommendation at individual jurisdictions. This timeline encompasses several components, including EMA approval time, Joint Clinical Assessment (JCA) production time, JCA report publication to local HTA submission (submission gap), and subsequent HTA decision-making processes. Group 2 focused on the effectiveness of JCA, which is defined as the extent to which JCA outputs support national submissions for companies and inform national decision-making for HTA bodies. The results from the breakout discussions were presented back to the full group.

### 2.3. Data Processing and Analysis

Survey responses were manually tabulated in an Excel file and analyzed using descriptive statistics. Data were reported as total responses if there were 10 or more respondents, with rankings applied where relevant. The first author conducted a content analysis of free-text comments and open-ended responses to identify key themes. The constant comparative method was then applied, grouping similar themes and refining categories as new insights emerged. This iterative process ensured accurate pattern identification and proper categorization across respondents. The first author conducted a content analysis of the free-text comments and open-ended questions to identify key themes, followed by the constant comparative method. The second author reviewed the results to verify the phases and themes expressed by the study participants. The breakout discussion outcomes were summarized by the first author, using notes from the rapporteurs and meeting recordings.

## 3. Results

### 3.1. Survey Results

Thirteen out of the twenty pharmaceutical companies responded to the survey, resulting in a 65% response rate. This included the two companies that participated in the pilot.

Nine of the thirteen respondents are international pharmaceutical companies, ranking among the top ten globally by their levels of investment in research and development in 2023 [16], a key indicator of high innovation. As such, the respondents provide insights from a global perspective rather than focusing on a specific geographic region.

### 3.2. Internal Planning and Strategic Consideration

Companies were asked to rate their readiness for the HTAR on a scale of 0 to 10, where 0 indicates completely unprepared and 10 represents fully prepared. None of the surveyed companies rated themselves at either extreme. Most companies positioned their readiness just above the midpoint, with scores of 6 or 7. To prepare for the HTAR, companies reported undertaking various actions across processes, resources, policy/advocacy, and pilot initiatives (Table 1). Key steps included conducting assessments of pipeline products expected to undergo JCA in 2025 (92% of respondents), participating in HTA-related conferences, workshops, and training sessions (92%), and establishing internal task forces dedicated to HTAR preparation (85%). However, only half of the surveyed companies had conducted internal mock JCAs to identify potential challenges. This may be attributed to the timing of product launches. Readiness for HTAR preparation also depends on a company’s portfolio; companies without products expected to undergo the JCA process in 2025 may have a more relaxed stance, adopting an observational approach.

All surveyed companies reported that EMA submissions are expected to proceed as planned despite the impact of HTAR on EU regulatory strategies. However, concerns were raised regarding uncertainties in the JCA process, timelines, and the timely delivery of JCA reports. Companies emphasized the importance of internal joint strategic planning, integrated decision-making for evidence-generation plans, closer cross-functional collaboration, and enhanced internal awareness of HTAR as key considerations for developing products subject to JCA. The survey also revealed that approaches to global regulatory strategies and JCA submissions are likely to vary depending on the specific asset. In terms of preparing for the JCA dossier, 50% responded dossier preparation will be led by a global HTA team with input from local affiliates (e.g., comment, feedback); other respondents suggested alternative approaches, reflecting differences in organizational structures and strategies. However, the internal use of JCA reports is expected to differ based on the jurisdictions to which submissions are made. Respondents suggested that as internal discussions progress, there is a growing recognition of the need for companies to remain flexible and adapt their strategies based on practical experience with HTAR.

### 3.3. Measuring Success

**Figure 1:**
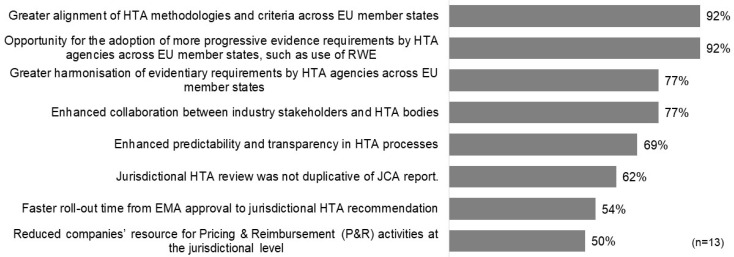
Potential long-term measures of the success of HTAR.

The surveyed companies offered diverse perspectives on how to define the success of the HTAR. In the short term, the majority highlighted key indicators of success as positive reimbursement decisions in EU jurisdictions (62%), non-duplicative jurisdictional HTA reviews aligned with the JCA report (54%), and enhanced predictability and transparency in HTA processes (54%). Interestingly, faster timelines from EMA submission to HTA recommendations were not considered a primary measure of success by any respondents. For long-term success, nearly all participants (92%) underscored the significance of achieving greater alignment of HTA methodologies and criteria across member states, along with the adoption of more progressive evidence requirements, such as real-world evidence (RWE), by EU HTA agencies (Figure 1).

The survey highlighted both internal and external challenges companies face in ensuring the timely integration of JCA reports into local HTA decision-making. Internally, the primary hurdles identified were difficulties in coordinating EU-level JCA processes with local HTA submission timelines (92% of respondents) and the complexity of aligning JCA findings with the specific requirements of individual jurisdictions (92%). To address these issues, respondents emphasized the importance of high-quality JCA submissions grounded in robust methodologies. Additionally, a “learning-by-doing” approach was recommended by a majority of respondents, supported by iterative feedback mechanisms and early planning during development to pinpoint assets and evidence requirements, leveraging JSC for guidance.

Externally, companies anticipated challenges such as the potential for inconsistent acceptance of JCA reports among HTA agencies (92% of respondents), duplicative submission processes and national-level requirements (92%), resource constraints within HTA agencies (77%), and the lack of established HTA systems in certain jurisdictions (77%). To address these issues, respondents proposed enhancing transparency and quality in both the JCA and national decision-making processes, promoting proactive agency engagement with companies during the JCA process, and refining the JCA process through iterative learning and continuous improvement.

### 3.4. Workshop Discussion and Recommendations

The break-out sessions were attended by 20 experts in Group 1 and 21 experts in Group 2, representing a diverse mix of stakeholders. The key insights and recommendations are summarized in Table 2.

**Figure 2:**
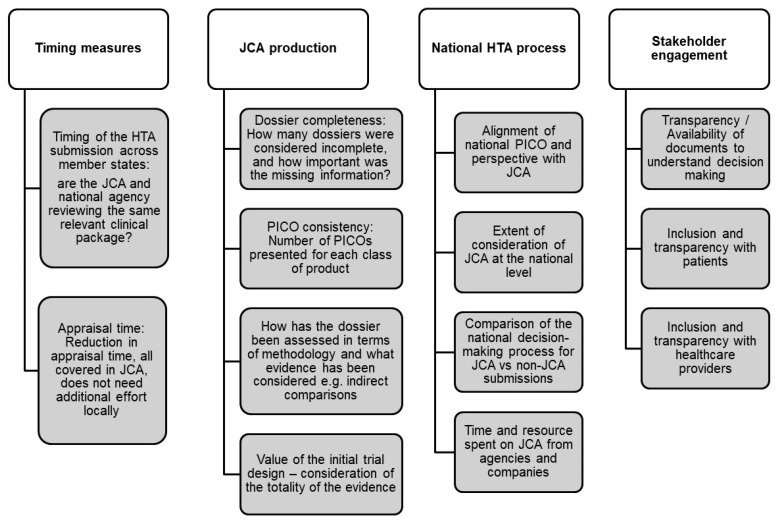
Develop metric framework/scorecard to evaluate efficiency and inform continuous improvement.

Both groups recommended developing essential metrics to ensure the effectiveness and efficiency of the JCA and national decision-making processes. This is particularly crucial for measuring time to market as a means to assess overall efficiency. For instance, key metrics include the timing of HTA submissions, which is important to ensure that both the JCA submission and national agencies are reviewing the same relevant clinical data. Additionally, the time to initiate the national submission process, the time required to validate a submission, and any changes over time are essential indicators. Regarding appraisal time, both groups emphasized reducing this duration, with the understanding that relevant clinical assessment should be covered within the JCA, thus avoiding the need for additional local efforts. Beyond timing, Group 1 further explored specific indicators to be incorporated into the metric framework (Figure 2).

The effectiveness of using JCA in national decision-making depends heavily on the quality and relevance of the JCA output. Group 2 agreed that the unclear role of JCA in decision-making processes across different countries is a key concern. To address this, stakeholders must develop a mutual understanding that the weight of the JCA in decision-making may vary by country. Effective communication should begin early and be tailored to different stakeholders, such as clinicians, patients, and HTA agencies outside the EU, to ensure clarity on what JCA output means for them.

Both the quality of the JCA submission and the quality of its review and output are critical. Group 2 recommended developing a product-based scorecard to evaluate each JCA dossier from the perspectives of various stakeholders. The proposed scorecard would take the form of a questionnaire to be completed by stakeholders, enabling companies to rate and provide feedback on the JCA review and output, while agencies could assess and provide feedback on the JCA dossier. The questionnaire could address key elements such as clear formatting; robust evidence in the submission, consistency, and predictability in the process; and the use of a sound methodology in the assessment. While the development of a detailed framework lies outside the scope of this discussion, the scorecard’s importance and potential value are recognized as foundational for monitoring quality improvements over time and fostering continuous improvement from both companies and agencies.

## 4. Discussion

This study provided an overview of companies’ readiness and strategic considerations for JCA, which are crucial for ensuring timely and high-quality submissions of JCA dossiers starting in January 2025. The stakeholder workshop, which was informed by the survey, expanded to include a broader range of multi-stakeholder perspectives. This approach went beyond the centralized JCA process, exploring the subsequent national roll-out and the JCA’s implications.

### 4.1. Time to Market—Not a Measure for Success but Critical to Ensure Efficiency

The time from EMA approval to jurisdictional HTA decision is a crucial metric for market access in Europe. For companies, speed to market directly correlates with the pace of return on investment; for HTA agencies, timely reviews and decisions demonstrate the efficiency of their processes; and for patients, swift recommendations enable access to new treatment options. Currently, the time to HTA decision varies significantly across European jurisdictions. The 2024 CIRS report highlighted that Germany has the fastest time, with only 156 days from EMA approval to HTA recommendation, while Poland had the longest time in the study, taking 529 days in 2023. However, the time between EMA approval and HTA decision is influenced by several factors, including the company’s submission strategy, pre-submission preparation time, and the HTA agency’s review process [6]. Notably, despite Poland’s long overall duration, the HTA review itself took a median of only 83 days in 2023. The extended timeline can primarily be attributed to delays in submission from EMA approval to the Polish Agency AOMiT.

While time to market is important to companies, our survey did not identify it as the key success factor for the HTAR. From a company perspective, a positive recommendation is considered more critical, as it is directly linked to market access. However, the hope is that the timely production of JCA reports shortly after EMA approval will help minimize submission delays in certain European countries. This was one of the key recommendations from the workshop discussions: reducing submission gaps and ensuring synchronized submissions to all HTA agencies across member states. Such alignment would ensure that agencies are reviewing similar clinical evidence, improving overall efficiency and consistency. Our survey revealed that companies are actively restructuring and preparing for both JCA and national submissions. The internal utilization of JCA reports is anticipated to vary depending on the target jurisdictions for submission. Consequently, aligning local affiliates to ensure timely submissions is a critical effort required from companies to ensure the potential efficiency gains by the JCA process.

The appraisal times at HTA agencies have been assessed across various jurisdictions in Europe [6]. Differences in remit, assessment criteria, and evidentiary requirements contribute to the divergences among agencies. A study by Akehurst highlighted that varying decision criteria influence both the timing and outcomes of HTA decisions. For example, France prioritizes disease severity, Italy focuses on efficacy, the Netherlands considers cost-effectiveness, long-term outcomes, and disease severity, while Germany emphasizes robust evidence and efficacy [17]. With the introduction of the JCA, the aim is to harmonize clinical assessments across Europe, focusing on policy questions rather than subjective value judgments. It can be assumed that, after agencies invest resources and efforts to evaluate a new product at the JCA level, national decision-making will be underpinned by the JCA outcome, thereby reducing duplication and saving time. Our workshop recommended measuring the time and resources spent at the national level, comparing the appraisal times for both JCA and non-JCA products to assess potential efficiency gains. However, it was also suggested that a learning process should be allowed as the new system is implemented. The initial JCA reviews and subsequent national reviews may take longer, but as the system evolves, appraisal times should reflect the efficiency gains brought by the JCA process. In addition, it is important for all jurisdictions to provide transparent explanations regarding their consideration of the JCA within their process, even if this is to clarify why it has not been utilized.

To build on previous research into measuring the rollout time of new medicines, a detailed milestone benchmarking framework should be established. This would provide insights not only into the overall time to market but also into each component of the process and where time is allocated. Key components to consider for measurement include the following: the time taken for JCA preparation, submission, and review; the time required for EMA submission and approval; the interval from JCA outcomes to national HTA submission; the appraisal time from national HTA submission to decision; and the time from national HTA decision to reimbursement. Additionally, capturing the timing between submissions to various HTA agencies is essential to evaluate whether the HTAR enables a more aligned approach. Currently, not all HTA agencies publish their review timelines, and research has primarily been conducted among agencies that disclose such data. To ensure that patients and other stakeholders can fully understand the efficiency of the JCA process, greater transparency in publishing both JCA outcomes and the national decision-making processes is necessary.

### 4.2. Improvement of Quality—Using Scorecard Framework to Ensure Iterative Improvement

The 21 EUnetHTA deliverables on “PICO without submission” explored the potential number of PICOs required for JCA across three products. The results revealed that Pluvicto required a six-PICO framework, Ebvallo needed a five-PICO framework, and Pombiliti required a nine-PICO framework [18]. As a result, companies have raised concerns about the number of PICOs required for the JCA process, as well as their relevance to national decision-making. To assess the necessity and effectiveness of the PICO framework, our study recommends implementing measures that evaluate PICO consistency, including the number of PICOs required for products within the same class, the alignment between national PICO requirements and JCA standards, and the extent to which JCA is considered at the national level. As a real-world example of the JCA report is not yet in place, experts in our workshop identified a key barrier: the unclear relevance of the JCA in decision-making. There is a need for mutual understanding and clear communication regarding the varying weight the JCA will hold in different decisions across countries.

To maximize the value of the JCA output, it is crucial to ensure the quality of the entire process, from the construction of the dossier to the JCA report and, ultimately, to national decision-making. The workshop recommended developing a product-based scorecard to assess both the quality of JCA submissions from companies and the quality of JCA reviews from agencies. The concept of a scorecard has been previously tested in the regulatory field, which consisted of over 50 items organized into seven key domains: application format, dossier content, labeling, scientific advice, review conduct, communication, and overall assessment. Studies showed that such a rating framework offers valuable insights into the differing perceptions of quality regarding submitted data and their respective review processes [19,20]. The findings demonstrated the value and applicability of the proposed scorecards in drug evaluation. The development of a scorecard involved a collaborative, multi-stakeholder approach. To establish a systematic process and a structured scorecard for regulatory review, active participation from both companies and regulatory agencies was essential. The scorecard system was designed based on best practices for the industry and good review practices for regulatory authorities, utilizing a methodology informed by a literature review and expert input. The process started from conceptualization to item generation, revision, and final scorecard development and validation. Feedback from scorecard exercises, conducted using actual product reviews, systematically identified areas where the review process excelled and highlighted opportunities for improvement [18].

Considering the importance of the newly introduced JCA process, we recommend that authorities and companies collaborate to develop a scorecard framework. Such a framework would enhance consistency in the JCA process and provide a structured mechanism for collecting feedback from all stakeholders. Although implementing this approach will require significant time and effort, it has the potential to serve as a vital tool for fostering continuous improvement in the JCA process.

### 4.3. A Learning Journey That Involves All Relevant Stakeholders

The JCA under HTAR represents the most significant and impactful collaboration among HTA agencies in Europe. HTA collaboration is expected to enhance the credibility and quality of HTA services and findings, drive efficiency gains, foster the development of joint methodologies, and promote alignment across agencies [21]. However, the process is unlikely to be perfect from the beginning. The HTAi policy forum discussions on collaboration emphasized that this process should involve iterative learning, with its impact measured against the primary objectives [22].

All workshop attendees endorsed a collaborative “learning-by-doing” approach to the implementation of JCA, acknowledging that learning from both successes and challenges is equally important. While HTA agencies are already exchanging knowledge through the HTA Coordination Group, it was suggested that companies also consider the development of open-source platforms, such as statistical tools, to reduce duplication and build capacity across the industry. In addition, local experts, including clinicians, patients, and patient organizations, should be actively involved in the JCA process.

Similar experiences and lessons can be drawn from the creation and evolution of the EMA. Established to harmonize the work of existing national medicine regulatory bodies, EMA launched its first centralized procedure for human medicines in 1995. As the regulatory landscape evolved, the Heads of Medicines Agencies (HMA) introduced the Benchmarking of European Medicines Agencies (BEMA) program in 2004 to assess the systems and processes of individual agencies across Europe against a set of established indicators. This benchmarking process helped identify areas of strength within regulators and highlighted opportunities for improvement. Over time, the EMA’s remit has also expanded, its role formerly considered as a gatekeeper of innovation, though now as an enabler of it. This evolution reflects the EMA’s increasing focus on advancing the development and approval of new medicines [21].

Similarly, the measures and scorecard framework proposed in the study could serve as a baseline to support the evolution of the JCA, ensuring it remains fit for purpose and both efficient and effective. It is hoped that the HTAR will serve not only its primary goal of harmonizing the clinical aspects of new medicines, but also foster broader advancements. As reflected in our survey, one of the highest-rated long-term successes is the alignment of HTA methodology and criteria across EU member states, as well as opportunities for adopting more progressive evidence requirements, such as the use of Real-World Evidence (RWE).

## 5. Study Limitation

This study has two main limitations. First, the survey sample is predominantly composed of innovative companies, with limited representation from small and medium-sized enterprises (SMEs), which may benefit most from early guidance to better understand HTA requirements. Feedback from SMEs would further enrich the collective knowledge and shared learnings across the industry. Second, as the four implementation acts are released by December 2024, some of the discussions held in June 2024 may now be outdated. Consequently, some of the recommendations in this paper may already be under consideration by the coordination group and subgroups.

## 6. Conclusions

The implementation of the HTAR and the JCA process marks a significant step toward harmonizing HTA decision-making across Europe. However, the study identifies several key issues that must be addressed to ensure its efficiency and effectiveness. These include the need for greater clarity on the role of the JCA in national reimbursement decisions, the establishment of timeline metrics for the JCA process, national submissions, and appraisals, as well as the development of mechanisms to ensure the efficiency and quality of the JCA process. In addition, fostering collaboration among all stakeholders—HTA agencies, industry, and patient representatives—remains essential. The proposed product-based scorecard presents a promising framework for evaluating the quality of JCA submissions and reviews, potentially driving continuous improvement and greater consistency across jurisdictions. Furthermore, a collaborative “learning-by-doing” approach, involving all stakeholders and prioritizing early engagement, will be crucial for navigating the evolving HTA landscape. As agencies and companies adapt to the new system, a focus on capacity-building, transparent communication, and iterative learning will be essential. This can be achieved through feedback studies on the quality of assessments and submissions, and timeline metrics which will help maximize the impact of the JCA process and ensure timely access to innovative products for patients across Europe.

## Figures and Tables

**Table 1:** Internal preparations for the EU HTA Regulation: actions taken by the companies.

Areas	Internal Actions	Respondents % (n = 13)
Process	Redesigned internal processes and identified resource gaps for HTA readiness	69%
	Reviewed and aligned internal policies and procedures for HTA regulation	38%
	Reviewed the internal structure of EU pricing and reimbursement mechanisms	38%
Resource	Established an internal task force dedicated to HTA regulation	85%
	Increase internal resource dedicated to HTA regulation	62%
Policy and Advocacy	Participated in HTA-related conferences, workshops, or training sessions	92%
	Active participation in advocacy efforts such public consultation, stakeholder network	85%
	Provided training and awareness initiatives across internal EU affiliates	77%
Pilot and Assessment	Conducted assessments of the pipeline products anticipated to undergo JCA in 2025	92%
	Conducted internal pilot studies or mock assessments to understand potential challenges	54%
	Participated in EUnetHTA rapid effectiveness assessments (EU REAs) pilots	38%

**Table 2:** Multi-stakeholder discussion recommendations.

Breakout Group 1 To Ensure Efficiency of the Processes from JCA to National HTA Decision Making	Breakout Group 2 To Ensure Effectiveness of Using JCA in National HTA Decision Making
Develop metrics framework/scorecard to evaluate efficiency and inform continuous improvement	Develop a product-based scorecard to evaluate each submission from different stakeholder perspectives (industry, agency, patient etc). Did the submission include the information each stakeholder needed and how did they rate the process?
Establish training programmes to support capability and capacity building for Member States.	Identify metrics that can help HTA agencies to understand the value of JSC and enable iterative improvement.
Facilitate open dialogue amongst Member States to share technology-specific learnings; what is needed through the JCA process and what are the considerations locally across Member States?	Enhance communication between stakeholders: more frequent and earlier communication. This must help to set expectations on what the JCA is and is not.
Develop open-source tools to reduce duplication and build capacity across health technology developers e.g. statistical tools.	Conduct research to understand what agencies expect from the JCA and how they will use the JCA report in their decision making. Agencies could be grouped by archetype to identify trends.

## Data Availability

The original contributions presented in this study are included in the article. Further inquiries can be directed to the corresponding author(s).
